# Comparison of three different self-expanding metal stents using rabbit models for the treatment of tracheal collapse

**DOI:** 10.1590/acb370502

**Published:** 2022-08-12

**Authors:** Ji-Hyun Kim, Jin-Young Choi, Hun-Young Yoon

**Affiliations:** 1 DVM., PhD. Konkuk University – College of Veterinary Medicine – Department of Veterinary Surgery – Seoul, South Korea.; 2 DVM. Konkuk University – College of Veterinary Medicine – Department of Veterinary Surgery – Seoul, South Korea.; 3 DVM., PhD. Konkuk University – College of Veterinary Medicine – KU Center for Animal Blood Medical Science – Department of Veterinary Surgery – Seoul, South Korea.

**Keywords:** Self Expandable Metallic Stents, Trachea, Rabbits

## Abstract

**Purpose:**

To identify an optimal self-expandable metallic stent (SEMS) and verify whether a mechanically superior SEMS would result in better clinical outcomes in the treatment of tracheal collapse.

**Methods:**

We selected three SEMS (n = 8 each), including an S-type stent with a wire diameter of 0.006 inches (S6) and two D-type stents with wire diameters of 0.006 (D6) and 0.007 inches (D7). Twenty-four New Zealand White rabbits were divided into three equal groups. After the stents were deployed, the clinical signs were recorded daily, and radiographic examinations were performed monthly. All rabbits were euthanized after three months.

**Results:**

Two rabbits with S6 stents and one rabbit with a D7 stent died within three months because of stent migration or pneumonia. All rabbits with D6 stents survived for three months. On histological examination, the D6 group had the lowest inflammation score.

**Conclusions:**

Both clinically and histopathologically, the results with D-type stents with a wire diameter of 0.006 inches were superior to those of the other groups (p = 0.001). The use of an optimal intraluminal stent may improve the long-term clinical outcomes in the treatment of tracheal collapse in dogs.

## Introduction

The mechanical properties of self-expanding metal stents (SEMSs), such as radial force (RF), axial force (AF), foreshortening ratio, reconstrainability, and flexibility, have been previously studied by numerous researchers due to their effect on clinical performances^1^^-^ 3. RF and AF are two main properties that are particularly emphasized by clinicians, as RF determines the extent to which the lumen must be expanded and maintained, and AF provides the flexural rigidity of the stent^2^. In human literature, studies have shown that a low AF yields better clinical outcomes with the use of SEMSs for coronary arteries and biliary obstruction^1^^,^ 4.

Tracheal collapse in dogs is a progressive condition that can result in life-threatening respiratory diseases, eventually leading to respiratory distress and sudden death. Since the procedure is quick, requires minimally invasive techniques, and covers the entire scope of tracheal lesions, the recent application of intraluminal stents is preferred while treating refractory tracheal collapse^5^. Various stents have been used in veterinary literature as a treatment for tracheal collapse in dogs^6^^-^ 8. Most SEMSs used in veterinary medicine are manufactured for human medicine, and the results of stent applications often lead to serious complications, including stent migration, stent fracture, and pneumonia^9^. Since the tracheas of humans and dogs have different anatomical characteristics, it is necessary to develop a stent specific for dogs. Recently, Vet Stent-Trachea (Infiniti Medical LCCTM, Malibu, CA, United States of America), a nitinol stent designed specifically for veterinary use, has become commercially available and it is commonly used to treat tracheal collapse^10^. However, a thorough evaluation of the mechanical properties of SEMSs used in veterinary medicine is still lacking.

In a previous study, the mechanical properties of SEMS with various mesh designs were compared. A double-wire woven SEMS with unfixed cells (D-type stent) had a higher RF and lower AF than a single-wire woven SEMS with fixed cells (S-type stent)^11^. Therefore, D-type stents have mechanical properties superior to those of S-type stents. In addition, both RF and AF increased significantly as the wire diameter of the D-type stents increased by 0.001 inches. In another study, a rabbit model of tracheal collapse was successfully created, and the mechanical properties of rabbits and dogs with respect to the trachea were compared. According to the results from previous chapters, two types of SEMS were selected: a D-type stent with a wire diameter of 0.006 inches (D6); and a D-type stent with a wire diameter of 0.007 inches (D7). Since the only commercially available and most used tracheal stent in veterinary medicine is the Vet Stent-Trachea, which is a single-wire woven stent with fixed cells, the S-type stent with a wire diameter of 0.006 inches (S6) was included in this study for clinical comparison.

The purposes of this study were to identify an optimal SEMS in terms of mechanical properties and wire diameter and to verify whether a mechanically superior SEMS would result in better clinical outcomes in the treatment of tracheal collapse.

## Methods

### Animals and stent types

This study was approved by the Institutional Animal Care and Use Committee of Konkuk University (IACUC NUMBER KU17026-1).

Twenty-four female New Zealand White rabbits weighing 3.5–4.0 kg were used to create experimental animal models of tracheal collapse. A radiographic examination was performed to eliminate any underlying diseases related to the trachea.

General anesthesia was administered intramuscularly using a combination of ketamine (35 mg/kg), xylazine (5 mg/kg), and butorphanol (0.1 mg/kg). The study animals were divided into three groups of eight each:

- Group A: S-type stent (wire diameter, 0.006 inches);
- Group B: D-type stent (wire diameter, 0.006 inches);
- Group C: D-type stent (wire diameter, 0.007 inches).

Postoperatively, enrofloxacin (10 mg/kg) and butorphanol (0.1 mg/kg) were administered subcutaneously for a week.

**Figure 1:**
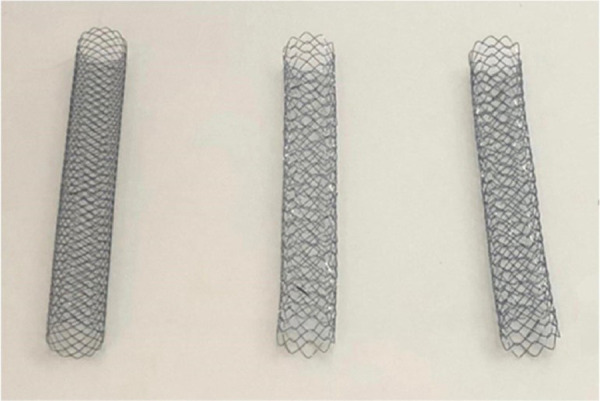
Fully deployed self-expanding metal stents. From left to right: S-type stent (wire diameter, 0.006 inches), D-type stent (wire diameter, 0.006 inches), and D-type stent (wire diameter, 0.007 inches).

Three different SEMS were selected based on the results of a previous study (Fig. 1). Eight S-type stents with wire diameters of 0.006 inches (S6), D-type stents with wire diameters of 0.006 inches (D6), and D-type stents with wire diameters of 0.007 inches (D7) were manufactured. The length of the delivery system was kept short for better manipulation through the trachea.

### Surgical procedure

**Figure 2:**
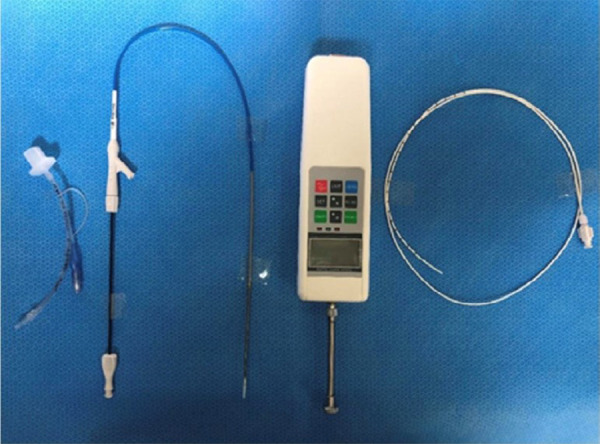
Equipment used for tracheal stenting in this study. From left to right: a 3-0 endotracheal tube, a self-expanding stent in the delivery system (S-type stent with a wire diameter of 0.006 inches, a D-type stent with a wire diameter of 0.006 inches,or a D-type stent with a wire diameter of 0.007 inches), a digital force gauge, and a 5-Fr radiopaque marker catheter.

**Figure 3:**
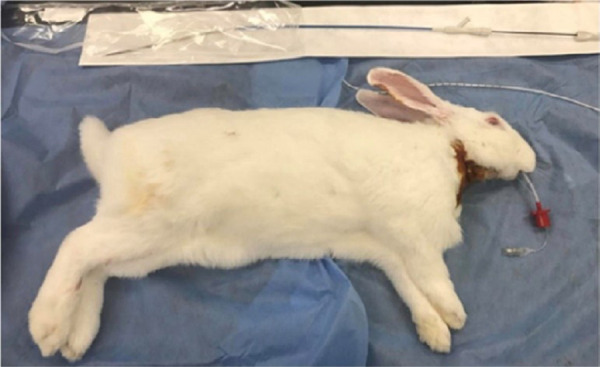
Preparation of a rabbit model for stent deployment. The rabbit model was intubated with a 3-0endotracheal tube, and the rabbit was positioned in lateral recumbency with the neck flexed.

**Figure 4:**
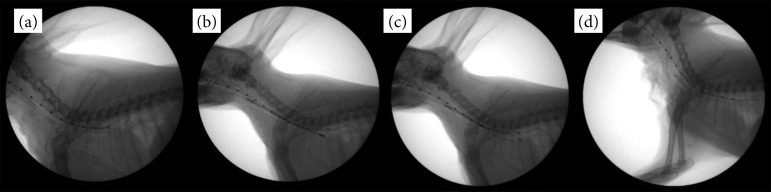
Fluoroscopic images of step-by-step stenting procedures. **(a)** A 5-Fr radiopaque marker catheter was inserted into the esophagus under fluoroscopic guidance. **(b)** Each delivery system was passed through the endotracheal tube, and the starting point of the stent was visualized using fluoroscopy. **(c)** The self-expanding metal stent was deployed slowly to ensure that the stent was positioned accurately. **(d)** Each stent was fully deployed, and the delivery system was carefully removed from the trachea.

Tracheal collapse was surgically induced in all rabbits. After general anesthesia, the rabbits were positioned in dorsal recumbency, clipped from the caudal mandibular area to the cranial thorax, and prepared for aseptic surgery. After exposing the cervical trachea, a length of 3 cm of tracheal cartilage, not penetrating the submucosal layer, was broken down systematically using a No. 10 blade. The RF of the trachea was measured using a digital force gauge (SH-200; Sundoo Instruments, Wenzhou, China) (Fig. 2). Each rabbit was positioned in lateral recumbency, with the neck flexed (Fig. 3). All rabbits were intubated using a 3-0 endotracheal tube (Medtronic, Dublin, Ireland). A 5-Fr radiopaque marker catheter (Cook Medical, Bloomington, IN, United States of America) was inserted into the esophagus under fluoroscopy as a guideline for accurate stent deployment (Fig. 4a). Each delivery system was then passed into the endotracheal tube, and the starting point of the stent was visualized using fluoroscopy (Fig. 4b). The SEMS was deployed slowly to ensure the accurate positioning of the stent (Fig. 4c). Each stent was fully deployed, and the delivery system was carefully removed from the trachea (Fig. 4d).

### Follow-up evaluation

All rabbits were clinically observed daily for three months, and any respiratory distress was recorded. Respiratory distress was assessed using the following grading of respiratory distress:

- 0 = normal, respiration without goose-honking sound;
- 1 = mild distress, intermittent goose-honking sound observed in excitement;
- 2 = moderate distress, intermittent goose-honking sound observed in rest;
- 3 = severe distress, continuous goose-honking sound observed;
- 4 = respiratory failure^12^.

Radiographs (TITAN 2000; Comed, Seongnam-Si, South Korea) were taken before and after surgery. Follow-up radiography was performed monthly for three months, and the diameters of the trachea before and after stent application at the surgical site were compared on radiographs and examined for signs of inflammation, stent migration, and stent fracture. The animals were euthanized three months after the surgery.

### Histological findings

After euthanasia, the tracheal samples from all rabbits were harvested from the cricoid cartilage to the thoracic inlet. Tracheal swabs were collected from the inside of each trachea for bacterial culture. The tracheal samples were fixed in 10% buffered formalin for 48 h, washed for 30 min, and decalcified in a decalcifying solution (Decalcifying Solution-Lite; Sigma-Aldrich, St. Louis, MO, United States of America) for 6 h. After washing for 30 min, the tracheal samples were fixed in 10% buffered formalin for 17 h. The same procedure was repeated the following day. The specimens were sectioned at 4-?m thickness and stained with hematoxylin and eosin. The degree of inflammation was evaluated based on the identification of polymorphonuclear neutrophils, lymphocytes, plasma cells, macrophages, giant cells, necrosis, neovascularization, fibrosis, and fatty infiltration. The number of the mentioned categories observed at the magnification of 400× was converted to a score. The inflammation score index was calculated as previously described^13^.

### Statistical analysis

Statistical analyses were performed using Statistical Package for the Social Sciences (SPSS) version 24 (IBM, Armonk, NY, United States of America). One-way analysis of variance was used for multiple comparisons, followed by a post-hoc Tukey (Tukey–Kramer) test. For non-parametric variables, the Kruskal–Wallis’ test was used for multiple comparisons, followed by the Mann–Whitney’s test. Statistical significance was established at p < 0.05.

The correlation between RF and weight was analyzed using a simple correlation analysis. Pearson correlation coefficients (r) > 0.7, > 0.5, and > 0.3 were considered as strong, moderate, and weak positive linear correlations, respectively. Statistical significance was established at p < 0.05.

## Results

### Clinical evaluation

**Figure 5:**
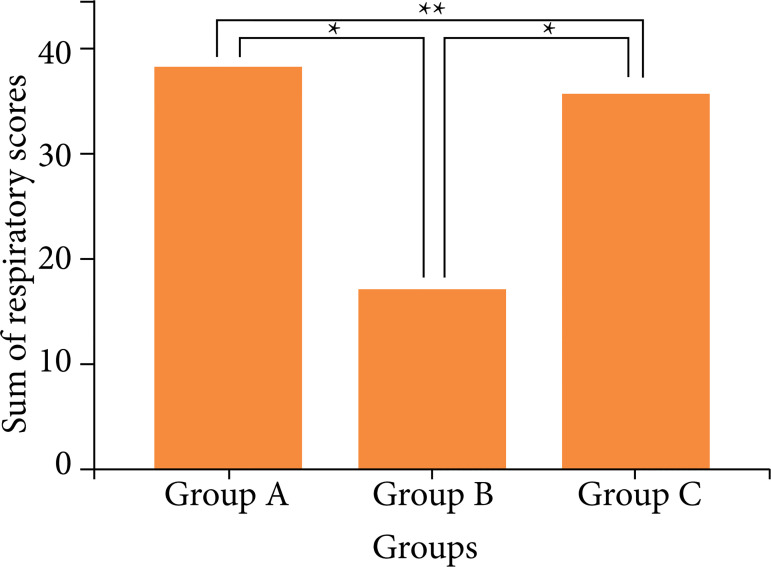
Sum of respiratory scores for each group. Group B had the lowest respiratory score.

All rabbits were monitored daily for three months. Two rabbits from group A died due to stent migration or pneumonia, and one rabbit from group C died due to pneumonia. All eight rabbits in group B were euthanized on schedule. The mean monthly respiratory scores were added to obtain the total score for each rabbit. The total scores were presented as mean ± standard deviation (SD). The total respiratory scores were 37.9 ± 2.83, 17.2 ± 0.72, and 33.8 ± 1.76 for groups A, B, and C, respectively. Group B had the lowest score in the clinical evaluation (p < 0.05) (Fig. 5). There were significant differences between groups A and B and between groups B and C (p < 0.05), although there were no significant differences between groups A and C (p > 0.05).

*p < 0.05, **p > 0.05.

### Radiographic examination

**Figure 6:**
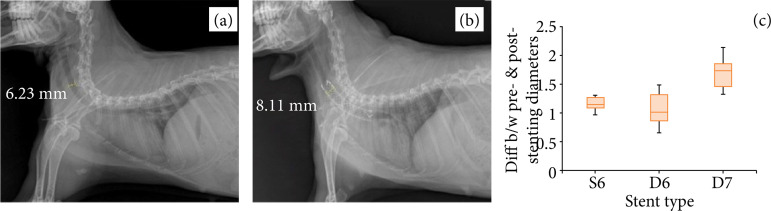
Lateral radiographic views of the trachea in a rabbit model before and after stenting. **(a)** The pre-stenting diameter of the normal cervical trachea. **(b)** The post-stenting (D-type with a wire diameter of 0.007 inches)diameter of the cervical trachea. **(c)** Boxplot of differences between the pre- and post-stentingdiameters (mm) for groups A, B, and C. The difference was the greatest in group C (p<0.05).

Pre- and post-stenting diameters were compared using radiography. Pre-stenting radiographs were obtained before the induction of tracheal collapse. The difference between the pre- and post-stenting diameters was calculated after calibration (Fig. 6). The differences were 0.62 ± 0.09, 0.61 ± 0.10, and 1.70 ± 0.24 mm for groups A, B, and C, respectively. The difference was the greatest in group C (p < 0.05). There were no significant differences between groups A and B (p > 0.05). Furthermore, significant differences were identified between groups A and C and between groups B and C (p = 0.000).

**Figure 7:**
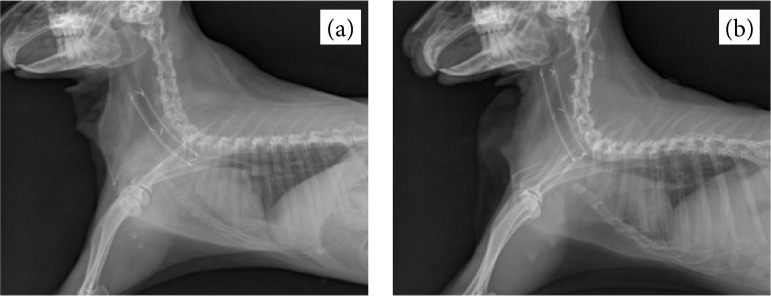
Lateral radiographic views of the trachea in a rabbit model before and after stentmigration. **(a)** An S-type stent with a wire diameter of 0.006 inches was placed in thetrachea of a rabbit. **(b)** Proximal stent migration resulting in respiratory failure.

Any signs of stent migration, fracture, tracheitis, or pneumonia were evaluated using radiographs. A major migration greater than 5 mm in measurement was only observed in one rabbit from group A, which led to respiratory failure (Fig. 7).

### Gross examination

**Figure 8:**
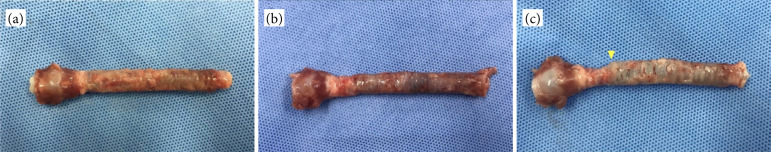
Harvested tracheal samples of rabbits with each type of self-expanding metal stent. **(a)** The S-type stent with a wire diameter of 0.006 inches. **(b)** The D-type stent with a wire diameter of 0.006 inches. **(c)** The D-type stent with a wire diameter of 0.007 inches. The tracheal diameters of **(a)** and **(b)** were consistent throughout the trachea. The yellow arrowhead in **(c)** indicates the area in which the tracheal cartilage is stretched due to the high radial force of the stent.

Surgically induced tracheal collapse in the cervical region was not observed in any rabbit after SEMS application. Tracheal diameters from samples of groups A and B were macroscopically consistent throughout the trachea (Figs. 8a and 8b). However, in group C, the tracheal diameters in the stenting area with D7 were greater than those in the non-stenting area. The tracheal diameters in the stenting area were stretched beyond their original tracheal diameters (Fig. 8c).

### Histological findings

**Figure 9:**
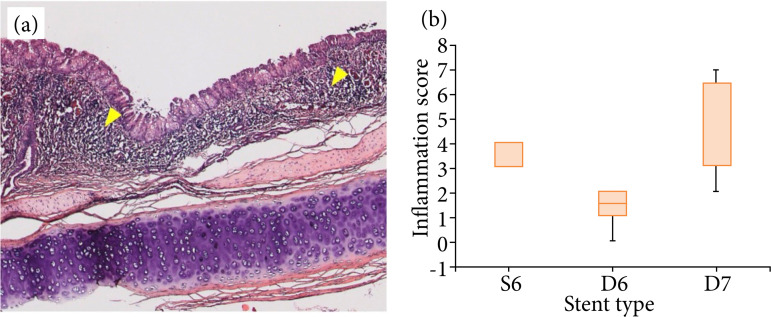
Inflammation around the stent insertion site. **(a)** Infiltration of inflammatory cells was observed in the submucosal layer after stent implantation in group A (Yellow arrow heads). **(b)** Boxplot of inflammationscores of the stented trachea. The inflammation score was the lowest in group B.

Each specimen was scored based on the presence of polymorphonuclear neutrophils, lymphocytes, plasma cells, macrophages, giant cells, necrosis, neovascularization, fibrosis, and fatty infiltration. Histological findings of granulation were observed in one rabbit in Group C. No significant granulation was observed in other rabbits; only inflammatory changes were observed. The sums of the inflammation scores were 4.5 ± 2.5, 1.4 ± 0.7, and 5.3 ± 4.3 for groups A, B and C, respectively. There were significant differences between groups A and B and between groups B and C (p = 0.000 and p = 0.001, respectively). No significant differences were identified between groups A and C (p > 0.05). The inflammation score was the lowest in group B (Fig. 9a, yellow arrow heads).

### Correlations between radial force and weights

**Figure 10:**
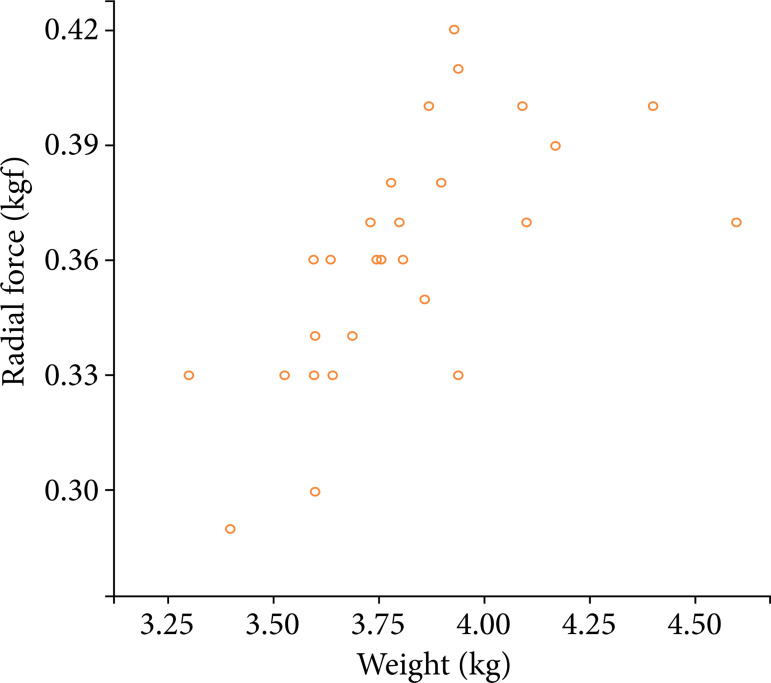
Linear correlation between the tracheal radial force of the rabbits and weight. Amoderate positive correlation was observed (r = 0.596, p = 0.002, and R^2^ = 0.355).

Positive or negative linear correlations between the normal tracheal RF of rabbits and weights in 24 rabbits were assessed using simple correlation analyses. There was a moderate positive correlation between the normal tracheal RF of rabbits and weight (r = 0.596, p = 0.002, and R^2^ = 0.355) (Fig. 10).

## Discussion

The exact causes of tracheal collapse in dogs remains unknown. Chondromalacia occurs as the supportive capacity of the tracheal cartilage weakens. As the laxity of the tracheal dorsalis muscle increases, the trachea becomes dorsoventrally flattened, and the cartilage eventually collapses^14^^,^ 15. The cervical trachea is exposed to atmospheric pressure, and the intrathoracic region is exposed to pleural pressure^16^. Cervical tracheal collapse occurs commonly in dogs. Rabbits have longer tracheas, particularly in the cervical region; therefore, they are excellent candidates for creating animal models for researchers^17^.

The placement of extraluminal rings and the placement of intraluminal stents are two surgical techniques commonly performed to treat tracheal collapse in dogs^18^. Complications have been reported with both techniques; however, fewer complications, application in any region of the trachea, non-invasiveness and short surgery, anesthesia, and recovery times are the reasons that stenting has become a more common treatment^19^^,^ 20. With advanced training and techniques, avoidable errors can be prevented, thereby lowering the complication rates after tracheal stenting^21^. Unavoidable complications may occur due to the mechanical properties of SEMS, dynamics of the trachea, and progression of tracheal collapse.

Numerous studies in human medicine have suggested that SEMS with a low AF yields better clinical outcomes^1^^,^ 2 ^,^ 4. When applying SEMS as a treatment for tracheal collapse, the most common complications were stent fractures, stent migration, and inflammation^10^. In this study, the post-stenting clinical signs, radiography, and microscopic findings were compared between rabbit models of tracheal collapse after inserting SEMS with different mechanical properties, including RF and AF. During clinical evaluation, two of the eight rabbits from group A died of respiratory failure due to stent migration and pneumonia. One of the eight rabbits from group C died of respiratory failure due to pneumonia. However, none of the eight rabbits from group B died before the termination of the study, and the evaluation of daily respiratory signs in this group was significantly better than that in the other groups. These results support the hypothesis that low AF would lead to better clinical outcomes in tracheal collapse as the AF of D6 was significantly lower than that of S6 and D7. Furthermore, SEMS with a low AF allows the stent to bend along the curvature, thereby causing less irrigation, inflammation, and migration. Therefore, a low AF may have led to a decreased risk of respiratory failure in group B.

Furthermore, stent migration occurred in only one rabbit in group A, and the result was associated with the high AF of S6. Since the AF of S6 was high, the tendency to straighten the tubular structure was stronger, thereby being more prone to migration^2^^,^ 22. In human literature, a significantly higher incidence of migration has been reported with the use of SEMS with higher AF for biliary obstruction^2^.

Another factor highly associated with stent migration is appropriate sizing. Owing to the dynamics of the trachea, lateral thoracic radiographs may not represent the maximal tracheal diameter^23^. In addition, the muscle tone of the trachea increases when a dog is awake, which may cause undersizing of the maximal tracheal diameter. Therefore, positive-pressure ventilation is needed to fully expand the trachea under general anesthesia^23^. However, this procedure was omitted in this study when measuring the maximal tracheal diameter because of the anatomical differences in the trachea between dogs and rabbits. The proportion of tracheal tracts in the total circumference of the trachea is less than 10% in rabbits; therefore, the variation in tracheal diameter with positive ventilation was assumed to be less^24^. Undersizing of the tracheal diameter may lead to stent migration; however, oversizing is also not recommended because lesser expansion of SEMS may exert excessive pressure on the tracheal wall^10^. Therefore, the stent diameter is typically chosen to be either 10–20% or 2–3 mm larger than the maximum tracheal diameter^19^^,^ 23. The stent diameter was selected to be approximately 2 mm larger than the maximum tracheal diameter of the rabbits.

In the comparison of microscopic findings among the groups, the inflammatory score was higher in groups A and C. Lower inflammation in group B may be the cause of better clinical outcomes. Furthermore, bacterial tracheitis was the most recorded complication in dogs after intraluminal stenting, and severe infection may lead to pneumonia^23^. Implanting a tracheal stent would cause a reaction that can result in inflammation; therefore, continuous monitoring is required in post-stenting patients. Nakai *et al*.^4^ reported that SEMS with high AF caused significantly more pancreatitis and cholangitis treated for biliary obstruction. The results from this study also demonstrated that the higher the AF of the SEMS, greater was the inflammation observed. In group C, all SEMS were fully expanded beyond their tracheal diameters. The tracheal cartilage rings were stretched on gross examination, and the differences between the pre- and post-stenting tracheal diameters were the greatest in group C, resulting from the high RF of the D7 stent. Even with an increase of 0.001 inches in the wire diameter of the SEMS, RF increased significantly. If RF is too strong, the pressure exerted on the trachea by the stent may lead to pressure necrosis or perforation of the trachea, thereby resulting in pneumomediastinum or pneumothorax^10^. Necrosis of the tracheal walls was not observed; however, the inflammation score was significantly higher in groups C and A than in group B. Kirsch *et al*.^24^ reported that the high RF of stents implanted in canine models of the carotid arteries stretched the inner layer of the vessel, thereby resulting in increased neointimal hyperplasia due to an increase in localized inflammation.

The most common isolates from bacterial cultures after intraluminal stentings in dogs are *Pasteurella multocida*, ?-hemolytic *Streptococcus* spp., and non-fermentative Gram-negative rods^23^. *P. multocida* was isolated from both rabbits that died from pneumonia in groups A and C. Not all tracheal samples were positive cultures, thereby suggesting that tracheal stenting was not always associated with infection. Even with infection, adequate antibiotic therapy could have prevented respiratory failure due to pneumonia. However, no additional antibiotics were administered to maintain the consistency of comparison.

Despite various case reports after applying SEMS in dogs with severe tracheal collapse, the association between tracheal RF and weight has never been discussed in existing studies^4^^,^ 23. In another study, a strong positive correlation between tracheal RF and weight was identified, which suggested that a greater RF may be required to maintain the collapsed lumen of the trachea in medium- to large-breed dogs. A moderate positive correlation was identified between the tracheal RF and weights of 24 rabbits in this study; however, the minimal requirement of RF to maintain the lumen would not vary because the variation in rabbit weights is less than the variation in dog weight. Moreover, because tracheal collapse has been reported even in large-breed dogs, the tracheal RF of the non-collapsed region would be greater than that of toy- and small-breed dogs. Owing to the compromised rigidity of tracheal cartilage rings in dogs with tracheal collapse, the RF of the collapsed tracheal region would be identically low. When applying SEMS as a treatment, the length of the stent is selected to span the maximal length of the trachea, including the non-collapsed region of the trachea^8^^,^ 23. Recurrence of clinical signs has been documented in the use of segmental stents in the collapsed tracheal region; theoretically, material fatigue would be increased on the segmental stents, which may lead to stent failure^10^. Therefore, to prevent further progression of the disease and firmly fix the stent to the tracheal mucosa, it is recommended that the stent be inserted from the cricoid cartilage to the carina with 1- to 2-cm safety margins of the trachea; moreover, the RF must be sufficiently high to exert adequate outward force to maintain the tracheal lumen from the non-collapsed region.

A limitation of this study is that it cannot establish a situation consistent with that of the actual diseased trachea. In general, dogs with tracheal stents, chronic cough, and chronic tracheitis have had these symptoms persisting for a long time, and underlying diseases often coexist (*i.e.*, bronchial collapse and heart disease). We created an animal model of tracheal collapse by surgically narrowing the tracheal lumen and reproduced the clinical signs of the disease. In this study, long-term results of more than three months were not confirmed in experimentally induced tracheal collapsed rabbit model; thus, the evaluation of the side effects of long-term application was limited. Therefore, long-term prognostic evaluation is required before application in cases of dogs with tracheal collapse. Further studies and clinical trials are needed to determine whether the same results as those of the preclinical test will be obtained in actual patients with worse cases.

## Conclusions

In this study, three different SEMS were compared in rabbit models of tracheal collapse. The results with a D6 stent with high RF and low AF were superior to those with an S6 stent with low RF and high AF and with a D7 stent with high RF and high AF in clinical evaluation, radiographic examination, and microscopic evaluation. The results of this study were favorable and consistent with the theoretical expectations based on the results of previous studies. Successful management of tracheal collapse with a D6 stent has been reported in dogs, and more clinical data will be available soon^5^. Therefore, from a clinical standpoint, stents with an easily expandable and more bendable character are most suitable and recommended for the treatment of tracheal collapse.
